# Supplementary material for: The fracture resistance of 3D-printed versus milled provisional crowns: An in vitro study
Source: PLoS One. 2023 Sep 1;18(9):e0285760. doi: 10.1371/journal.pone.0285760 (PMC10473469; doi:10.1371/journal.pone.0285760)
Supplement: S1 File — (PDF) [file pone.0285760.s001.pdf]

The results obtained from the series of experiments were then transferred to an Excel spreadsheet and prepared for statistical analysis. This was then performed using the statistical software SPSS, version 24.0 (IBM, New York, USA).

The following table shows the results of the test series: Table 1: Overview of the test results

Sample No.	Group	Sample designation	Fmax N	dL at Fmax mm	F at break N	dL at break mm
1	0	Preliminary test 11	525	3,4	515	3,4
2	1	No.1 16 SmileTemp	1120	1,8	1120	1,8
3	1	No.2 16 SmileTemp	1190	1,9	1140	1,9
4	1	No.3 16 SmileTemp	1470	2,0	1440	2,0
5	1	No.4 16 SmileTemp	1080	1,9	1050	1,9
6	1	No.5 16 SmileTemp	1250	1,8	1250	1,8
7	1	No.6 16 SmileTemp	1550	1,7	1550	1,7
8	1	No.7 16 SmileTemp*	727	1,2	726	1,2
9	1	No.8 16 SmileTemp	1240	1,7	1240	1,7
10	2	No.1 16 CADTemp	1560	1,9	1520	1,9
11	2	No.2 16 CADTemp	1610	1,5	1590	1,5
12	2	No.3 16 CADTemp	1990	2,1	1990	2,1
13	2	No.4 16 CADTemp	2130	1,9	2110	1,9
14	2	No.5 16 CADTemp	1540	1,6	1540	1,6
15	2	No.6 16 CADTemp	1840	1,6	1740	1,6
16	2	No.7 16 CADTemp	2170	1,7	2170	1,7
17	2	No.8 16 CADTemp	1960	1,7	1880	1,7

18	3	No.1 11 SmileTemp	309	2,0	308	2,0
19	3	No.2 11 SmileTemp	394	2,5	393	2,5
20	3	No.3 11 SmileTemp	647	1,9	647	1,9
21	3	No.4 11 SmileTemp	186	1,9	186	1,9
22	3	No.5 11 SmileTemp	229	1,8	229	1,8
23	3	No.6 11 SmileTemp	253	1,4	241	1,4
24	3	No.7 11 SmileTemp	321	1,5	313	1,5
25	3	No.8 11 SmileTemp	256	2,5	256	2,5
26	4	No.1 11 CADTemp	320	2,1	308	2,1
27	4	No.2 11 CADTemp	373	2,3	359	2,3
28	4	No.3 11 CADTemp	321	2,3	321	2,3
29	4	No.4 11 CADTemp	454	2,0	432	2,0
30	4	No.5 11 CADTemp	447	2,2	443	2,2
31	4	No.6 11 CADTemp	492	2,1	492	2,1
32	4	No.7 11 CADTemp	617	1,9	617	1,9
33	4	No.8 11 CADTemp	585	3,2	575	3,2

Source: own creation

The data from Table 1 were automatically displayed graphically in a curve diagram in the testXpert II software as previously described. However, since the tests were not linear, the results were presented in several diagrams for a better overview, as can be seen in Figures 21, 22, 23 and 24.

As previously described, the materials were always tested alternately in the main test series. For the subsequent statistical evaluation, the data were divided into four groups after successful completion of the test, as shown in Table 2.

Table 2: Overview of grouping

Group	Toot h	Material	Manufact urer
1	16	VarseoSmile Temp	Bego
2	16	VITA CAD-Temp monoColor	VITA
3	11	VarseoSmile Temp	Bego
4	11	VITA CAD-Temp monoColor	VITA

Source: own creation

The data from the four groups were then analyzed in descriptive statistics with reference to their mean (MW) and standard deviation (SD).

Table 3: Descriptive statistics of all groups

Group		Fmax N	dL at Fmax mm	F at break N	dL at break mm
1	MW	1203,38	1,750	1189,50	1,750
	SD	251,861	,2449	250,854	,2449
2	MW	1850,00	1,750	1817,50	1,750
	SD	253,659	,2000	258,222	,2000
3	MW	324,38	1,938	321,63	1,938
	SD	145,010	,4033	145,898	,4033
4	MW	451,13	2,263	443,38	2,263
	SD	111,716	,4033	113,632	,4033

Source: own creation

For better illustration, Table 3 has been presented in a bar chart.

Figure 20: Mean values and standard deviations of all groups

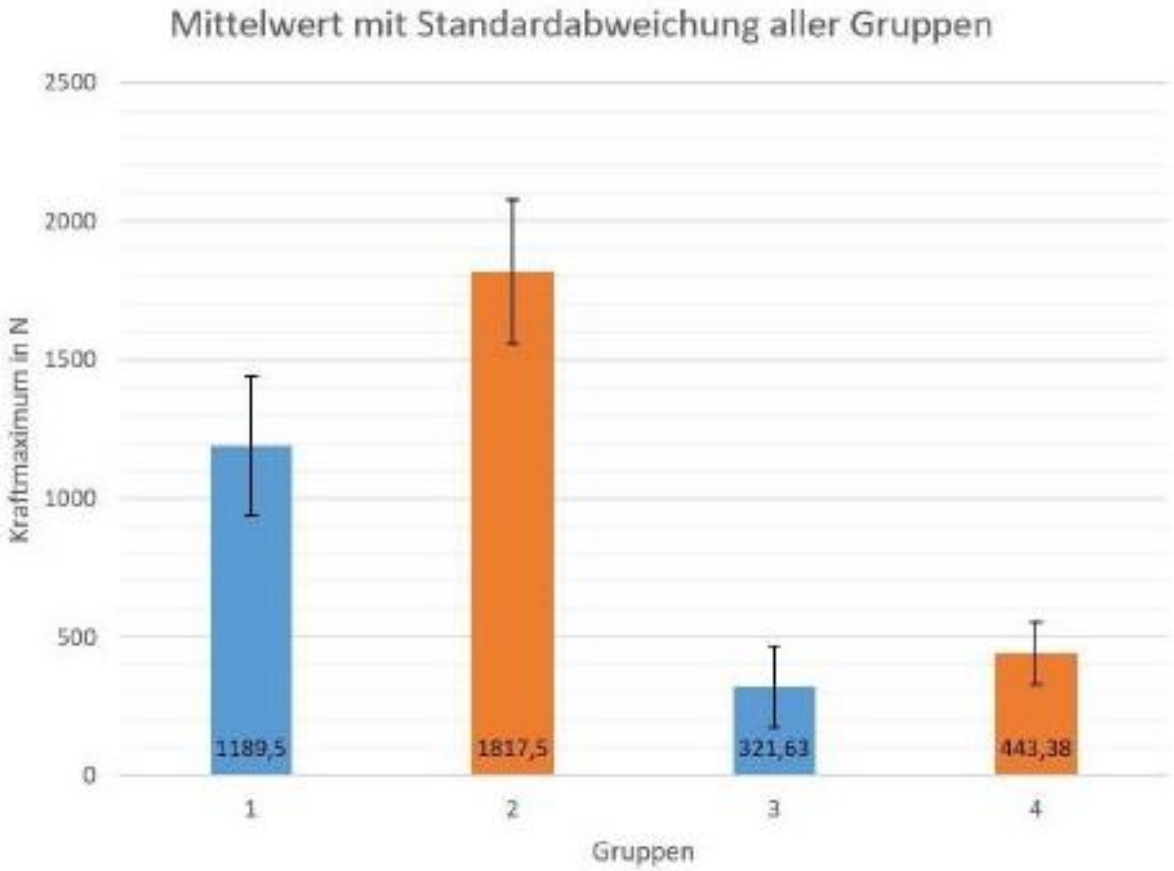

Source: own creation

The bars shown in Figure 20 represent the respective groups, the blue bars being the VarseoSmile Temp material and the orange bars being the VITA CAD-Temp monoColor material. As can already be seen here, the VITA CAD-Temp monoColor material was able to achieve higher mean values than the VarseoSmile Temp material both in the area of temporary restorations 16 and in the area of temporary restorations 11.

Temporaries 16 and 11 of the two materials were then compared. For this purpose, the T-test was determined using descriptive statistics. The main focus was on the key figure p-value (Sig. (2-sided)), which indicates the probability of the null hypothesis by a sample result with a value between zero and one. (Bender and Lange 2001) As a first group comparison, groups 1 and 2 were compared. These were the provisional 16 of the respective materials.

Table 4: Descriptive statistics of groups 1 vs. 2

	Group	N	MW	SD	Standard error of the mean
Fmax N	1	8	1203,38	251,861	89,046
	2	8	1850,00	253,659	89,682
dL at Fmax mm	1	8	1,750	,2449	,0866
	2	8	1,750	,2000	,0707
F at break N	1	8	1189,50	250,854	88,690
	2	8	1817,50	258,222	91,295
dL at break mm	1	8	1,750	,2449	,0866
	2	8	1,750	,2000	,0707

Source: own creation

Table 5: T-test from descriptive statistics, group 1 vs. 2.

			95% confidence interval of the difference				
	Mean difference	Standard error of the difference	At	Upper	T	df	Sig. (2-sided)
Fmax N	-646,625	126,381	-917,685	-375,565	-5,116	14	,000
dL at Fmax mm	,0000	,1118	-,2398	,2398	,000	14	1,000
F at break N	-628,000	127,282	-900,993	-355,007	-4,934	14	,000
dL at break mm	,0000	,1118	-,2398	,2398	,000	14	1,000

Source: own creation

Table 6: Descriptive statistics of groups 3 vs. 4

	Group	N	MW	SD	Standard error of the mean
Fmax N	3	8	324,38	145,010	51,269
	4	8	451,13	111,716	39,497
dL at Fmax mm	3	8	1,938	,4033	,1426
	4	8	2,263	,4033	,1426
F at break N	3	8	321,63	145,898	51,583
	4	8	443,38	113,632	40,175
dL at break mm	3	8	1,938	,4033	,1426
	4	8	2,263	,4033	,1426

Source: own creation

Table 7: T-test from descriptive statistics, group 3 vs. 4.

			95% confidence interval of the difference				
	Mean difference	Standard error of the difference	At	Upper	T	df	Sig. (2-sided)
Fmax N	-126,750	64,719	-265,558	12,058	-1,958	14	,070
dL at Fmax mm	-,3250	,2017	-,7575	,1075	-1,612	14	,129
F at break N	-121,750	65,382	-261,981	18,481	-1,862	14	,084
dL at break mm	-,3250	,2017	-,7575	,1075	-1,612	14	,129

Source: own creation

11 Appendix

Figure 21: Curve diagram of test series 16 VarseoSmile Temp

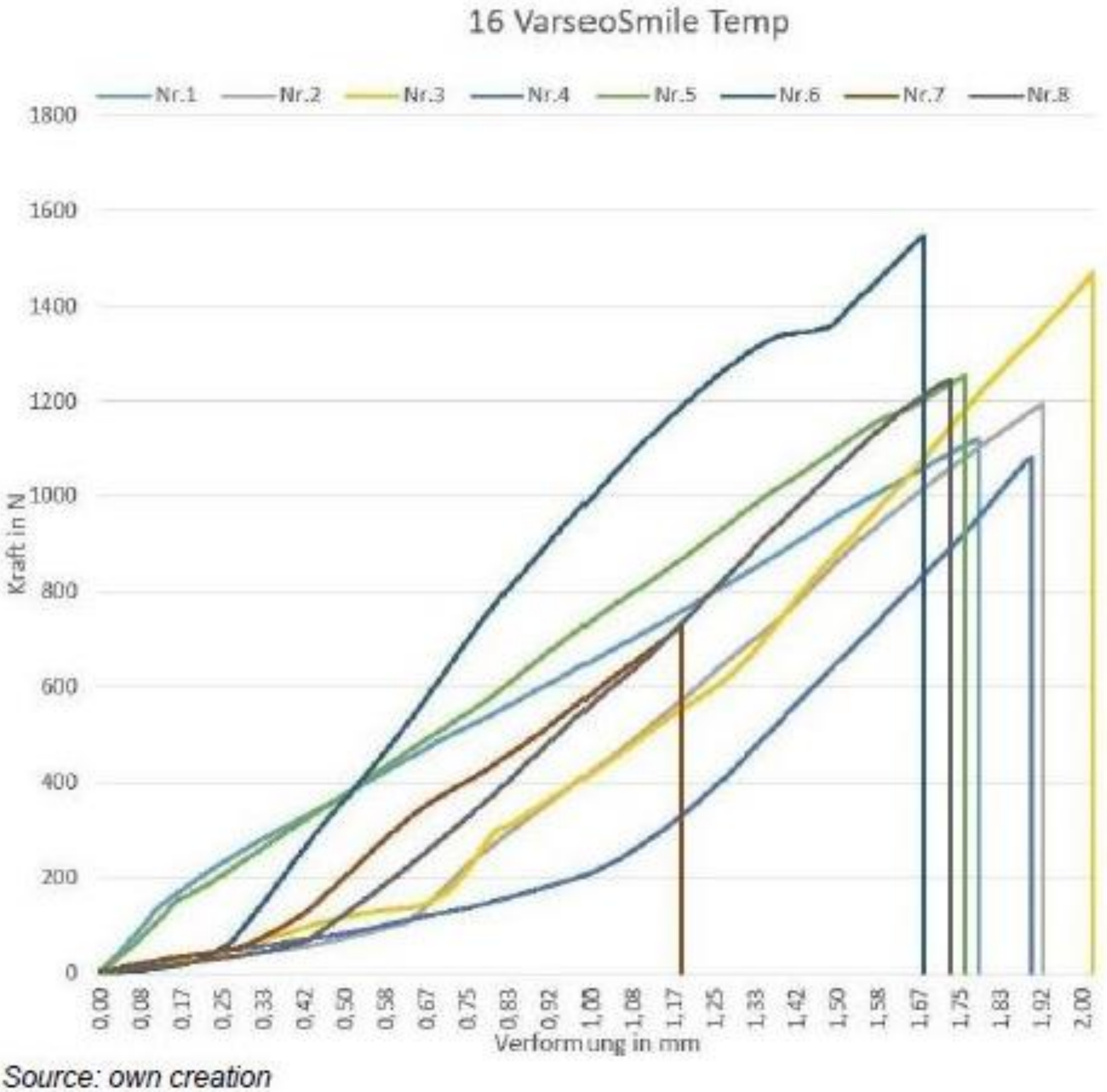

Source: own creation

Figure 22: Curve diagram of test series 16 CAD-Temp monoColor

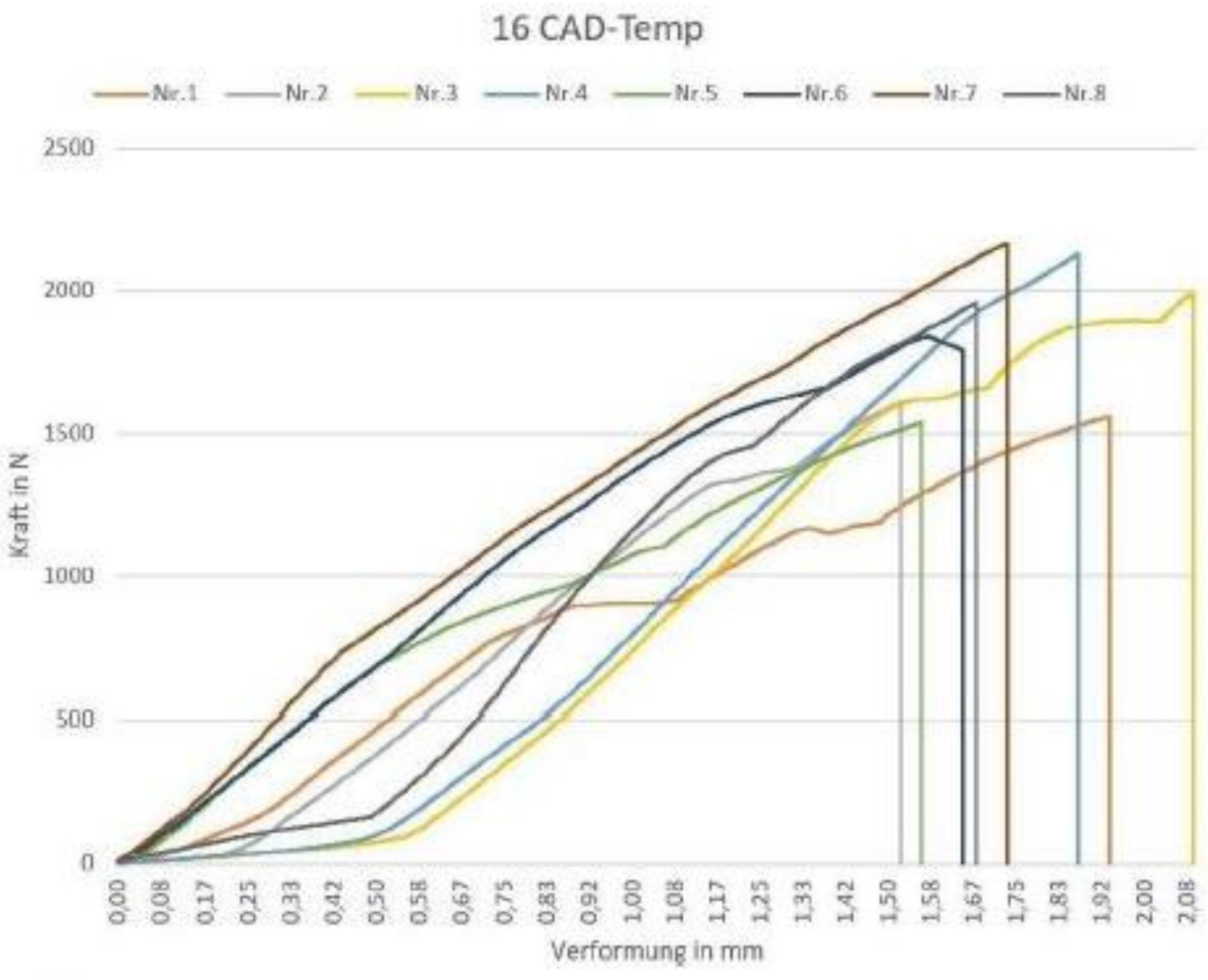

Source: own creation

Figure 23: Curve diagram of test series 11 VarseoSmile Temp

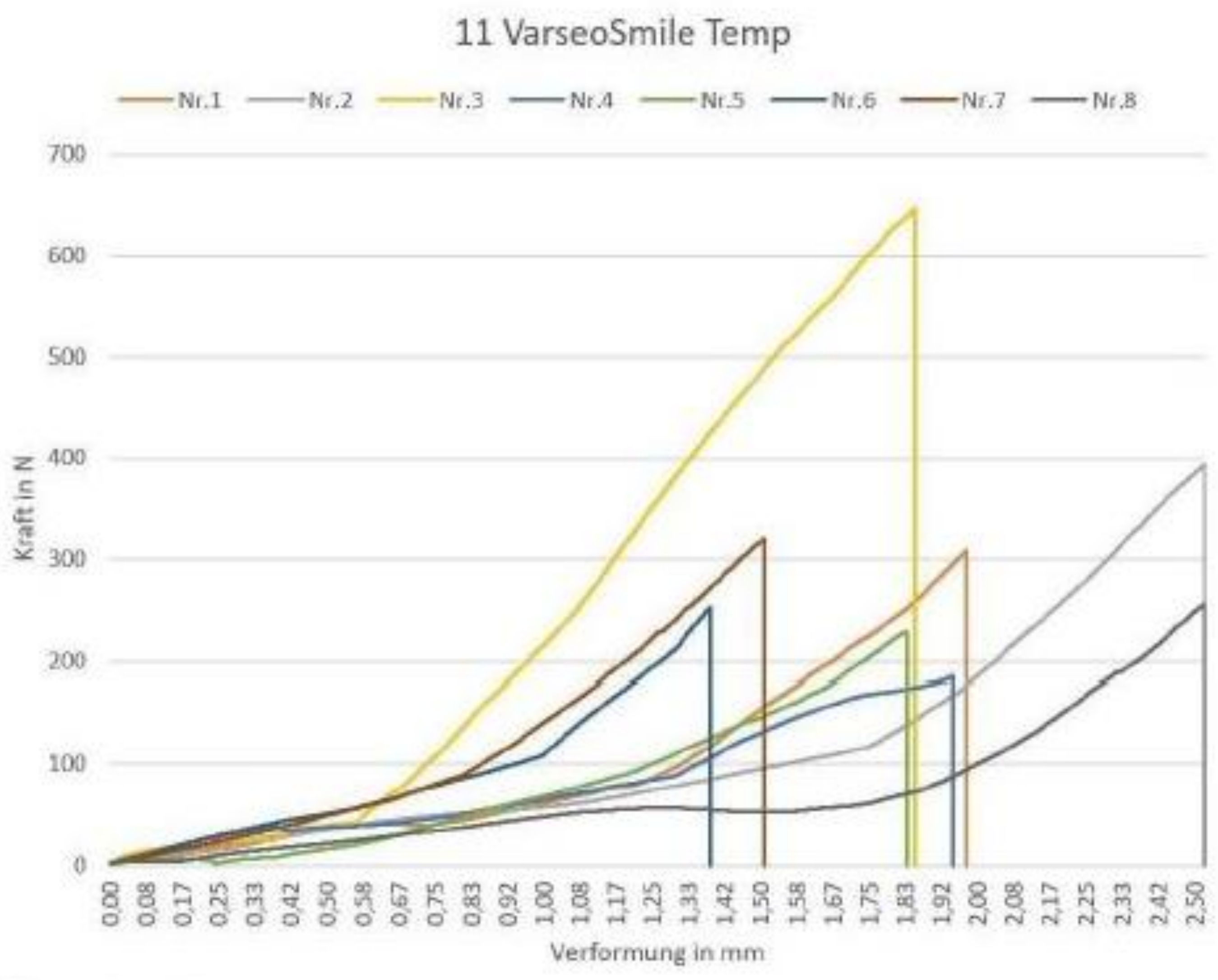

Source: own creation

Figure 24: Curve diagram of test series 11 CAD-Temp monoColor

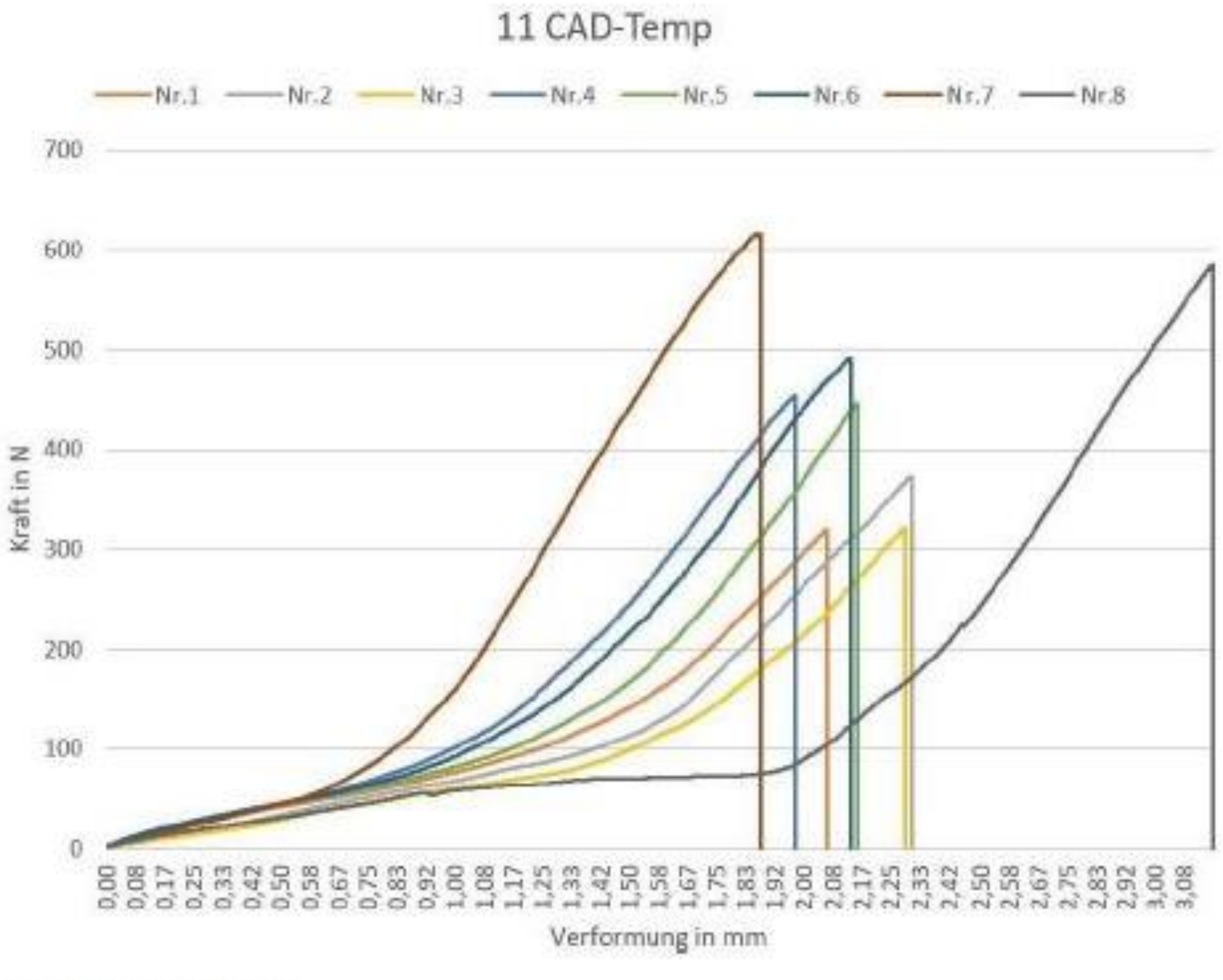

Source: own creation
