# Supplementary material for: The fracture resistance of 3D-printed versus milled provisional crowns: An in vitro study
Source: PLoS One. 2023 Sep 1;18(9):e0285760. doi: 10.1371/journal.pone.0285760 (PMC10473469; doi:10.1371/journal.pone.0285760)
Supplement: Attachment — Submitted filename: Responce to reviewers.docx [file pone.0285760.s002.docx]

December 20, 2022

Dear Dr. Mirza Rustum Baig,

Thank you for giving me the opportunity to submit a revised draft of my manuscript titled “**Comparative mechanical testing of 3D-printed versus milled provisional crowns: An in vitro study**" to the Journal PLOS ONE. We appreciate the time and effort you and the reviewers have dedicated to providing valuable feedback on the manuscript, responded appropriately as noted below, and highlighted the changes within the manuscript.

**Reviewer 1:**

This study aims to analyze the fracture resistance of implant-supported 3D-printed temporary crowns compared to milled crowns by compression testing. And they found that the milled molar group revealed significantly higher mechanical fracture strength than the 3D-printed molar group (P<0.001). However, no significant difference between the 3D-printed incisors and the milled incisors was found (P=0.084). Some comments.

1. Title. It is better to replace the “Comparative mechanical testing” in the title with more specific testing.

Response: Thank you for your guidance and advice. Done

“The fracture resistance of 3D-printed versus milled provisional crowns: An in vitro study”

2. Abstract. If possible, it is better to add brief on the printed and milling technique used. Which materials were used for printing implant crowns?

Response: Done

“The first group (16 specimens) was 3D printed by a mask printer (Varseo, BEGO, Bremen, Germany) with a temporary material (VarseoSmile Temp A3, BEGO, Bremen, Germany). The second group was milled in which a millable temporary material was used (VitaCAD Temp monocolor, Vita, Bad Säckingen, Germany).”

3. Introduction. The introduction is short. It will be better to add details on the Digital techniques of fabrication of crowns. Then explaining more on the 3D printing and milling technologies with indications, advantages and limitations. Some recent literatures are. <https://link.springer.com/chapter/10.1007/978-981-16-7152-4_12>

<https://www.ncbi.nlm.nih.gov/pmc/articles/PMC7356564/>

Response: Done

“Milled resin-composite crowns made using CAD/CAM have emerged as a potential alternative to metallic restorations in recent years [5]. However, some difficulties such as milling bar degradation, material waste, and stringent requirements for adequate abutment preparation must be emphasized. Therefore, three-dimensional printing is a promising, rapid, and cost-efficient method of creating dental prostheses digitally. It is a sophisticated manufacturing technology that use computer-aided design digital models to automatically generate personalized 3D objects [6]. Ceramics and resin are among the materials that be used in 3D printing. Digital light processing (DLP) and stereolithography (STL) provide speedy printing and good precision [7]. The DLP approach offers quick printing and excellent accuracy. The item is created according to the CAD design utilizing a resin-filled vat for layer-by-layer photopolymerization on the platform in the DLP process.”

4. Method. Please add on the sample size calculation.

Response: Done

“In the present investigation, 32 specimens were digitally designed using Aadva Software (GC, Tokyo, Japan) by estimation of the effect size 0.5 with 80% power (alpha = .05, two-tailed).”

5. Please discuss more on the results differences between the molars and incisors.

Response: Done

“If both test groups are considered from a mechanical point of view, the force acting on the molars is perpendicular, in contrast to the incisors, where the force acts at an angle of 45° - the tooth surface is negligible. In this case, the incisors are loaded differently than the molars, since the crown (force arm) and the acting force form a cross product, which acts as a torque on the rotational axis of the crown. Due to this lever, a greater force acts mechanically on the crown than is indicated on the machine. In the case of the molars, there is no torque because the force acts perpendicularly on the crown.”

**Reviewer 2:**

The aim of this study was to analyze the fracture resistance of implant-supported 3D-printed temporary crowns compared to milled crowns by compression testing.

1. As the full-length article, the Introduction section and the Discussion section were too short.

Response: Done

2. Only fracture loads are not enough.

Response: Thank you for your comment. We agree with you. We used the compression test which is One of the most used techniques. It is used to assess a material's behavior or reaction under crushing pressures, as well as to test a material's plastic flow behavior and ductile fracture limits. Compression tests are conducted by loading the test specimen between two plates and then applying a force to the specimen by moving the crossheads together. During the test, the specimen is compressed, and deformation versus the applied load is recorded. This study aims to analyze the fracture strength of implant-supported 3D-printed temporary crowns compared to milled crowns by compression testing. That would be addressed by measuring; first the fracture forces between the molar groups, Secondly the fracture forces between the incisor groups, Thirdly the deformation between the molar groups, and lastly the deformation between the incisor groups. Thus, it was possible to determine whether the materials differed in fracture resistance or deformation and whether there were differences within tooth regions. We hypothesized that the fracture strength of the 3D-printing method is non-inferior to that of the milling method.

3. Fatigue test and finite element analysis should be contained to strengthen this manuscript.

Response: Thank you for your comment. We agree, a finite element analysis would strengthen this manuscript. But also, without a finite element analysis reader can have clinically relevant and useful information from our manuscript.

**Reviewer 3:**

1. The abstract need revision.

Response: Sorry for the inconvenience. Done

“Abstract

Aims: This study aims to analyze the fracture resistance of implant-supported 3D-printed temporary crowns compared to milled crowns by compression testing.

Methods: The study sample included 32 specimens of temporary crowns which were divided into 16 specimens per group. Each group consisted of eight maxillary central incisor crowns (tooth 11) and eight maxillary molar crowns (tooth 16). The first group (16 specimens) was 3D printed by a mask printer (Varseo, BEGO, Bremen, Germany) with a temporary material (VarseoSmile Temp A3, BEGO, Bremen, Germany). The second group was milled in which a millable temporary material was used (VitaCAD Temp mono-color, Vita, Bad Säckingen, Germany). The two groups were compression tested until failure to estimate their fracture resistance. The loading forces and travel distance until failure were measured. The statistical analysis was performed using SPSS Version 24.0. We performed multiple t-tests and considered a significance level of p <0.05.

Results: The mean fracture force of the printed molars was 1189.50N (±250.85) with a deformation of 1.75mm (±0.25), whereas the milled molars reached 1817.50N (±258.22) with a deformation of 1.750mm (±0.20). The printed incisors fractured at 321.63N (±145.90) with a deformation of 1.94mm (±0.40) while the milled ones fractured at 443.38N (±113.63) with a deformation of 2.26mm (±0.40). The milled molar group revealed significantly higher mechanical fracture strength than the 3D-printed molar group (P<0.001). However, no significant difference between the 3D-printed incisors and the milled incisors was found (p=0.084). There was no significant difference in the travel distance until fracture for both the molar group (p=1.000) and the incisors (p=0.129). Conclusion: Within the limits of this in-vitro investigation, the printed and milled temporary crowns can both withstand the masticatory forces and are safe for clinical use.”

2. Materials and Methods need to be rewritten again.

Response: Done

3. Discussion need to include comparisons with similar previously published articles.

Response: Done

“The results in the present study are in line with previously published articles. The meta-analysis of Jain et al. compared articles comparing the physical and mechanical properties of 3D-printed provisional crown and FDP resin materials with CAD/CAM milled and conventional provisional resins. They concluded that that 3D-printed provisional crown and FDP resin materials have inferior physical properties compared to CAD/CAM milled ones (23). Furthermore, Ellakany et al. investigated the mechanical properties of CAD/CAM milled and two different types of 3D-printed, 3-unit IFDPs in comparison to the conventional IFDPs after the thermo-mechanical aging process. The conclusion of the study was that superior flexural strength, elastic modulus, and hardness were reported for milled IFDPs (24).”

4. References are not as per journal requirements.

Response: Thank you for the kind information. References style and format were checked to be following journal guidelines.

5. Sample size is not justified and very small. What is the power of this study???.

Response: thank you for your comment. In the present investigation, 32 specimens were digitally designed using Aadva Software (GC, Tokyo, Japan) by estimation of the effect size 0.5 with 80% power (alpha = .05, two-tailed). The 3D printer Varseo S (Bego, Bremen, Germany) and the milling unit inLab MC X5 (Sirona, Bensheim, Germany) were used to produce 16 specimens per group from the same digitally designed crowns. Each group consisted of eight maxillary central incisor crowns (tooth 11) and eight maxillary molar crowns (tooth 16).

6. What kind of Force was applied on incisor and molars, how much ???.

Response: Done

“For fracture resistance testing a load was applied at a cross-head speed of 0.5 mm/min and at the tooth until fracture occurred, according to ISO 11405/2003 (21).”

7. Conclusion should be revised. What is the mean fracture strength between the group of crowns for incisors and molars???

Response: Done

“Within the limitations of the present study, the fracture strength testing showed that milled molars crowns (1817.50N ±258.22) have statistically significantly higher fracture strength compared to 3D printed ones (1189.50N ±250.85), but in case of incisor crowns no statistically significant difference was shown in the present study (443.38N ±113.63 vs. 321.63N ±145.90). In all cases either crown shape and production method no statistically significant differences were shown in terms of deformation.

Regarding the results from a clinical point of view, those significant differences between the crowns in terms of fracture strength both can withstand the masticatory forces and are safe for clinical use.”
