# Supplementary material for: The fracture resistance of 3D-printed versus milled provisional crowns: An in vitro study
Source: PLoS One. 2023 Sep 1;18(9):e0285760. doi: 10.1371/journal.pone.0285760 (PMC10473469; doi:10.1371/journal.pone.0285760)
Supplement: Attachment — Submitted filename: Responce to reviewers.docx [file pone.0285760.s003.docx]

15.Feb.., 2023

Dear Dr. Mirza Rustum Baig,

Thank you for giving me the opportunity to submit a revised draft of my manuscript titled “**The fracture resistance of 3D-printed versus milled provisional crowns: An in vitro study**" to the Journal PLOS ONE. We appreciate the time and effort you and the reviewers have dedicated to providing valuable feedback on the manuscript, responded appropriately as noted below, and highlighted the changes within the manuscript.

**Reviewer 1:**

The authors have not addressed some previous comments.

Introduction. It will be better to add details on the Digital techniques of fabrication of crowns. Then explaining more on the 3D printing and milling technologies with indications, advantages and limitations. Some recent literatures are:

https://link.springer.com/chapter/10.1007/978-981-16-7152-4_12
https://www.ncbi.nlm.nih.gov/pmc/articles/PMC7356564/

1. The authors have not addressed some previous comments.

Response: We would like to apologize for any inconvenience. The missing comments have been precisely addressed as much as possible. Thank you for your guidance, advice and understanding.

2. Introduction. It will be better to add details on the Digital techniques of fabrication of crowns. Then explaining more on the 3D printing and milling technologies with indications, advantages and limitations.

Response: Done

“3D/4D printing can be integrated with artificial intelligence and machine learning to apply for patient-specific medical technologies (7). The recent digital techniques of fabrication of crowns includes Digital light processing (DLP) and stereolithography (STL) which provide speedy printing and good precision (8). The main advantages of CAD/CAM technologies includes accuracy, time-efficieny, doability and becoming a main part of healthcare technology solving complex medical problems which are promising for a rapid and economical technology for the digital fabrication of dental prostheses (7) They also provide superior mechanical strength, excellent esthetic and optical characteristics, and trustworthy precision and accuracy, which expand the clinical spectrum and allow for novel and less invasive restorative solutions (14). In terms of digital versus conventional workflow, the benefit lies in digital modeling and virtual planning, allowing several procedures to be performed with software and without human contact. The spread of infectious agents as COVID-19 can be more easily limited by reducing the number of work steps and procedures that can generate aerosols and environmental contaminants (15). The main limitations of CAD/CAM technologies include the high initial cost, the lack of color gradients in 3D-printed prostheses, technology failure, and the learning curve.

**Reviewer 3:**

Minor revision includes English language editing, providing complete statistics for assessment. kindly check image quality including resolution, pixels , blurring if any . I would appreciate more pictures from groups. Please add on clinical significance of this study. From my side as of now my queries had been met.

1. Minor revision includes English language editing, providing complete statistics for assessment.

Response: The English language editing was controlled by Springer Nature; certificate is attached for your preference. The statistics analysis for your kind assessment are attached as supplementary file.

2. kindly check image quality including resolution, pixels, blurring if any

Response: Done following Preflight Analysis and Conversion Engine (PACE) digital diagnostic tool, <https://pacev2.apexcovantage.com/>.

3. I would appreciate more pictures from groups.

Response: Done. More pictures were added in the article (Fig.1-3).

4. Please add on clinical significance of this study.

Response: Done. clinical significance was added in the article.

5. From my side as of now my queries had been met.

Response: Thank you for the kind understanding, advice and effort.

**Reviewer 4:**

The topic is interesting but unfortunately there are some important lacking. For example; The abstract is poor and not informative. The test groups are inadequate. Because there are different materials for this purpose. Are the crowns include screw hole or not? How does effect this feature to study? Generally, the information presented are not new, and does not add anything new to the current understanding. This study can't serve important clinical results.

1. The topic is interesting but unfortunately there are some important lacking. For example; The abstract is poor and not informative.

Response: Done. The abstract was modified to be more informative.

2. The test groups are inadequate. Because there are different materials for this purpose.

Response: Both milled and 3D printed materials must fulfill specific mechanical properties to withstand the occlusal masticatory forces and can be used clinically as durable temporary restorations as well as possibility for definitive restorations production. Both materials are prescribed as long-term temporary which can withstand mechanical forces. Accordingly, testing both materials for evaluating the comparative forces is considered reasonable. Especially that milled crowns are considered the gold-standard. Comparing printed material to the bench-mark milled material might be considered adequate.

3. Are the crowns include screw hole or not? How does effect this feature to study?

Response: They don’t include screw hole. The spacer thicknesses were between 30 and 50 μm were tested for both the anterior and posterior regions. The spacer settings of 40 μm ultimately produced the best results on the abutment, whereupon these were adopted for the anterior and posterior region. However, since for the milled temporaries the default standard settings in the Exocad software of 60 μm had been defined and the findings obtained for the printed temporaries could not be transferred to the milled temporaries. the two materials had to be produced with different spacer thicknesses. Current studies showed that in both 3D printing and milling, the difference between digital milling between the digital setting and the real result are sometimes enormous. differences occur in spacer thicknesses. (Hoang, Lisa N.; Thompson, Geoffrey A.; Cho, Seok-Hwan; Berzins, David W.; Ahn, Kwang Woo (2015): The spacer thickness reproduction for central incisor crown fabrication with combined computer-aided design and 3D printing technology: an in vitro study. In: The Journal of prosthetic dentistry 113 (5), S.398–404. DOI: 10.1016/j.prosdent.2014.11.004).

4. Generally, the information presented are not new, and does not add anything new to the current understanding. This study can't serve important clinical results.

Response: clinical use cannot be simulated entirely in vitro with a standardized test, it is possible to find material-specific properties in vitro, which are essential for its fundamental understanding. Various methods are available to analyze the mechanical strength of materials. One of the most used techniques is compression testing.
