# Supplementary material for: The fracture resistance of 3D-printed versus milled provisional crowns: An in vitro study
Source: PLoS One. 2023 Sep 1;18(9):e0285760. doi: 10.1371/journal.pone.0285760 (PMC10473469; doi:10.1371/journal.pone.0285760)
Supplement: Attachment — Submitted filename: Response to Reviewers.docx [file pone.0285760.s004.docx]

28.March.2023

Dear Dr. Mirza Rustum Baig,

Thank you for giving me the opportunity to submit a revised draft of my manuscript titled “**The fracture resistance of 3D-printed versus milled provisional crowns: An in vitro study**" (PONE-D-22-19019R2) to the Journal PLOS ONE. We appreciate the time and effort you and the reviewers have dedicated to providing valuable feedback on the manuscript, responded appropriately as noted below, and highlighted the changes within the manuscript.

**Journal Requirements:**

1) ‘’Please review your reference list to ensure that it is complete and correct. If you have cited papers that have been retracted, please include the rationale for doing so in the manuscript text, or remove these references and replace them with relevant current references. Any changes to the reference list should be mentioned in the rebuttal letter that accompanies your revised manuscript. If you need to cite a retracted article, indicate the article’s retracted status in the References list and also include a citation and full reference for the retraction notice’’.

**Response:** We would like to apologize for any inconvenient it might occur due to incorrect references list. Respectfully find in the manuscript with track changes the modified version of citations following the PLOS reference style outlined by the International Committee of Medical Journal Editors (ICMJE). The reference number 17 was eliminated. Also, reference number 7 was fully corrected. All other references were modified following “Vancouver” style. Thank you.
